# Computational Studies of a Mechanism for Binding and Drug Resistance in the Wild Type and Four Mutations of HIV-1 Protease with a GRL-0519 Inhibitor

**DOI:** 10.3390/ijms17060819

**Published:** 2016-05-27

**Authors:** Guodong Hu, Aijing Ma, Xianghua Dou, Liling Zhao, Jihua Wang

**Affiliations:** Shandong Provincial Key Laboratory of Biophysics, College of Physics and Electronic Information, Dezhou University, Dezhou 253023, China; maj667554ma@126.com (A.M.); dzxinyue2003@yahoo.com.cn (X.D.); zhaoll@sina.com (L.Z.)

**Keywords:** MD simulation, MM-PBSA, HIV-1 PR, drug resistance

## Abstract

Drug resistance of mutations in HIV-1 protease (PR) is the most severe challenge to the long-term efficacy of HIV-1 PR inhibitor in highly active antiretroviral therapy. To elucidate the molecular mechanism of drug resistance associated with mutations (D30N, I50V, I54M, and V82A) and inhibitor (GRL-0519) complexes, we have performed five molecular dynamics (MD) simulations and calculated the binding free energies using the molecular mechanics Poisson–Boltzmann surface area (MM-PBSA) method. The ranking of calculated binding free energies is in accordance with the experimental data. The free energy spectra of each residue and inhibitor interaction for all complexes show a similar binding model. Analysis based on the MD trajectories and contribution of each residues show that groups R2 and R3 mainly contribute van der Waals energies, while groups R1 and R4 contribute electrostatic interaction by hydrogen bonds. The drug resistance of D30N can be attributed to the decline in binding affinity of residues 28 and 29. The size of Val50 is smaller than Ile50 causes the residue to move, especially in chain A. The stable hydrophobic core, including the side chain of Ile54 in the wild type (WT) complex, became unstable in I54M because the side chain of Met54 is flexible with two alternative conformations. The binding affinity of Ala82 in V82A decreases relative to Val82 in WT. The present study could provide important guidance for the design of a potent new drug resisting the mutation inhibitors.

## 1. Introduction

Human immunodeficiency virus infection and acquired immune deficiency syndrome (HIV/AIDS) is caused by infection with the human immunodeficiency virus (HIV) [1]. The World Health Organization (WHO) estimated that there were about 35 million HIV-1 infections worldwide at the end of 2013 and an estimated 2.1 million individuals were newly infected in 2013. HIV-1 protease (PR) cleaves the viral precursor polypeptides Gag and Gag-Pol into mature, functional proteins, which is important for viral particle maturation [2]. Thus, HIV-1 PR has been an important target for anti-AIDS drug therapies. However, the drug-resistant mutations of PR continue to be a major problem in the treatment of AIDS [3].

HIV-1 PR is a homodimeric aspartic protease composed of a *C*_2_-symmetric dimer containing 99 amino acids in each chain. The dimerization interface forms the evolutionarily conserved catalytic triad, Asp25(Asp25′)-Thr26(Thr26′)-Gly27(Gly27′). Two ionizable residues (Asp25 and Asp25′) can interact directly with the inhibitor.

**Figure 1:**
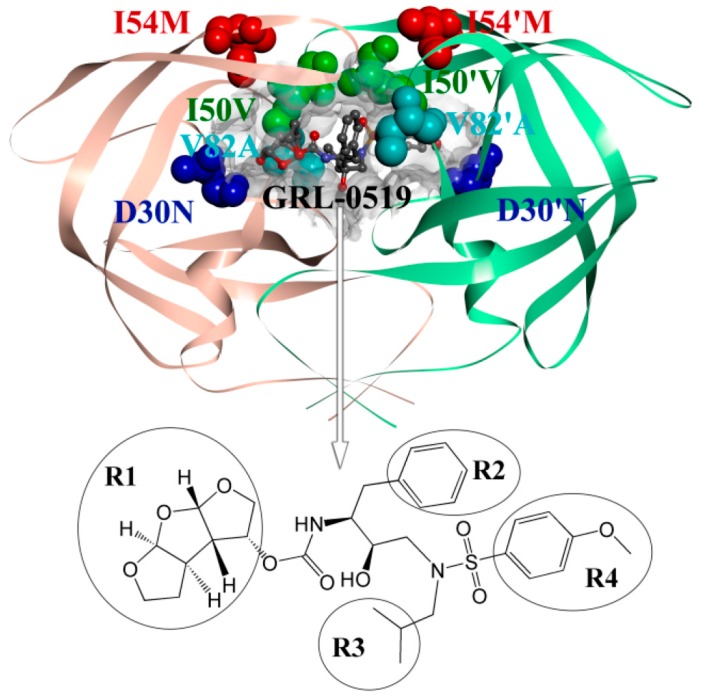
The locations of the four mutations in the wild type (WT). The PR is displayed in a solid ribbon representation, chain A is shown in light orange, chain B in pale green, GRL-0519 is displayed in a ball and stick representation, and the mutated residues are displayed as a ball with different colors as well as the labeled markers. The molecular structure of HIV-1 PR inhibitor GRL-0519 and the four groups (R1, R2, R3, and R4) are also shown.

Up to now, the Food and Drug Administration (FDA) has approved nine protease inhibitors for HIV therapy [4]. The current inhibitors showed initial success; unfortunately, the occurrence of drug-resistant mutations in the target enzyme caused by the high replication limited the effectiveness of these inhibitors [5]. Analysis of the biochemical and structural properties of PR mutations suggests that the mutations in the binding site can decrease binding affinities while maintaining the critical PR function in viral replication [6,7]. Two clinical protease inhibitors (PI) contain tetrahydrofuran (THF) in the R1 group. One inhibitor, darunavir (DRV) with bis-THF, introduces more hydrogen bonds with PR main chain atoms than the other one, amprenavir (APV), with a single THF. The DRV exhibits clinical efficacy and high potency against resistant viral infections [8,9]. A novel PI called GRL-0519 was synthesized (Figure 1) by Weber *et al.* [10]. GRL-0519 with the third THF in R1 fits better in the binding pocket of PR and shows large binding affinity [11].

More than 20 mutations of PR have been identified *in vitro* and *in vivo* [12,13]. Among all these mutations, D30N is associated with resistance to nelfinavir (NFV) [14]. D30N also alters the inhibitor binding in the PR-DRV complex. Mutations of residues Ile50 and Ile54, located at the flap, can affect the binding affinity for inhibitors for the variation of the conformational dynamics. The mutation V82A can decrease the binding affinity. In this work, we are interested in the drug resistance mechanics about D30N, I50V, I54M, and V82A mutations. The WT and GRL-0519 complex and the locations of mutated residues are shown in Figure 1.

Molecular dynamics (MD) simulations and free energy calculations were used to study the drug resistance mechanism underlying the binding of WT and its variants to GRL-0519. A few computational methods are used to estimate inhibitor binding affinities and selectivity with various levels of computational accuracy and expense. The molecular mechanics Poisson–Boltzmann surface area (MM-PBSA) method is useful for calculating the binding free energies of a given protein–inhibitor complex. In the MM-PSA, a series of representative snapshots from MD trajectories were collected to incorporate the effects of thermal averaging with force field/continuum solvent models. [15]. Compared with rigorous methods such as free energy perturbation (FEP), MM-PBSA is more computationally efficient [16].The MM-PBSA method was developed by Kollman *et al.* [17] and has been widely used ever since, with more than 100 publications each year from 2010 to now [18]. The performance of the MM-PBSA method varies depending on what system it is applied to [18]. This method has been used in conformer stability [19], protein–protein interactions [20,21], protein design [22], and re-scoring [23,24]. In several recent works, this method has also been successfully used to study multi-drug resistance in HIV-1 protease and inhibitor binding interactions [25,26,27,28,29,30,31].

To obtain information about the binding of GRL-0519 to WT and mutations, MD simulations have been successfully carried out to study the binding mechanics of GRL-0519 and WT and the molecular mechanism of drug resistance reduced by mutations D30N, I50V, I54M, and V82A. The contributions of different free energy components on GRL-0519 binding were decomposed by using the MM-PBSA method. A per-residue basis decomposition method was used to obtain the binding free energies between GRL-0519 and individual PR residue. The decomposed interaction energies provide insight into PR and GRL-0519’s binding mechanisms and help elucidate the drug-resistant mechanism of mutations to GRL-0519 with the interpretation of the energetic and structural results from the MD simulations.

## 2. Results and Discussion

### 2.1. The Flexibility of WT and Mutated Complexes

**Figure 2:**
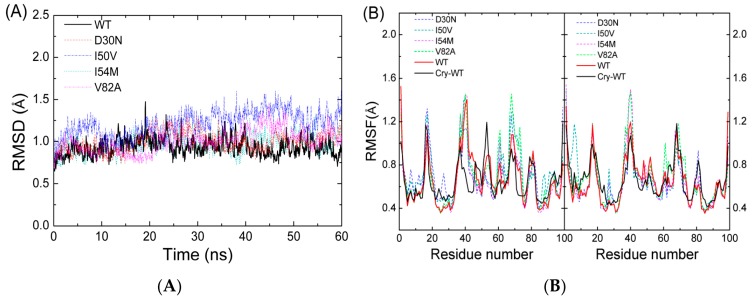

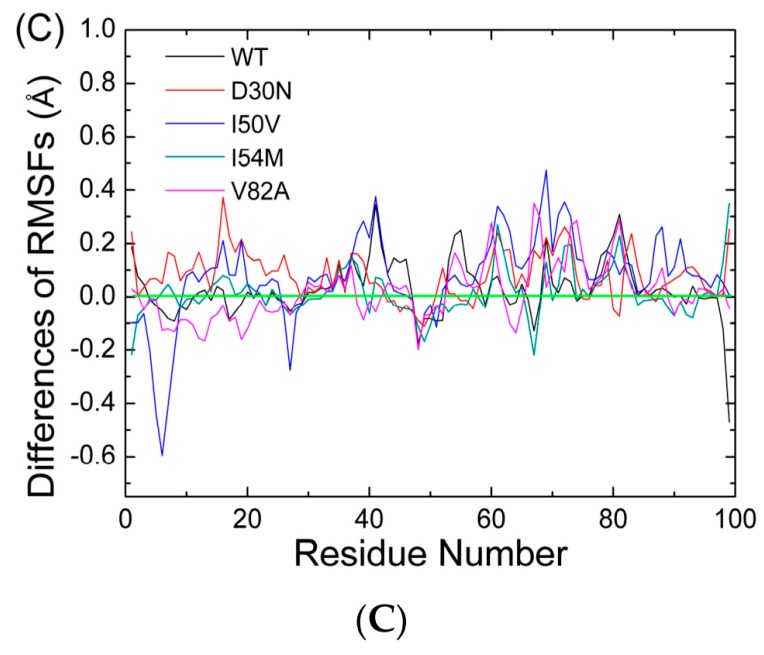
(**A**) The root mean square deviations (RMSDs) of the backbone atoms relative to their crystal structures as a function of MD simulation time; (**B**)the root mean square fluctuations (RMSFs) of backbone atoms *versus* residue number; (**C**) the differences of RMSFs values of the same residues in two chains *versus* residue number.

We carried out a 60-ns MD simulation for each complex. In order to assess the quality of our MD simulations, we monitored the structural and energetic properties during the whole MD simulation. The root mean square deviations (RMSDs) of the PR backbone atoms, which can reflect the stability of the complex, were analyzed relative to the corresponding starting structures of MD simulations for all complexes (see Figure 2A). It is clear that all systems have reached an equilibrated state after 5-ns MD simulations. We also calculated the RMSDs of the backbone atoms for the mutated systems relative to the starting structure of the WT complex (see Figure S1). As shown in Figure S1, the average RMSDs value of the WT system (0.94 Å) is slightly smaller than any one of the mutations during the last 40 ns of MD simulations. The last 40 ns of MD trajectories were also used in the subsequent analysis. The averaged RMSDs values of D30N, I50V, I54M, and V82A are 1.42, 1.21, 1.36, and 1.07 Å, respectively. This implies that the conformation of WT is slightly different from the four mutations in some regions, especially for D30N and I54M.

Furthermore, analysis of root mean square fluctuations (RMSFs) of backbone atoms *versus* the residue number for all complexes is illustrated in Figure 2B. Figure 2B also shows the experimental RMSFs of the WT complex obtained by transformation of the experimental B-factors, which is obtained from the crystallographic structure file, with the equation $\langle \Delta r_{i}^{2}\rangle =3B_{i}/(8\pi^{2})$. Large fluctuation was found in the flexible loop regions (residues 16–19/16′–19′, 35–42/35′–42′, 52–54/52′–54′, 66–69/66′–69′, and 78–81/78′–81′). For all complexes, a high degree of rigidity in catalytic site Asp25/Asp25′ was observed. This is in accordance with the function of catalytic site and Chen’s work [31]. From comparisons between the RMSFs in chain A and chain B, we noticed that the averaged RMSFs of chain A are larger than those of chain B for all complexes. The differences in RMSFs for each residue between chain A and chain B were calculated and shown in Figure 2C. The values for all complexes are smaller than zero in a region (around residue 50). This implies that the two chains have different models in this region. This is in accordance with the fact that the two chains of protease are *C*_2_-symmetry and the inhibitor is not (Figure 1).

**Figure 3:**
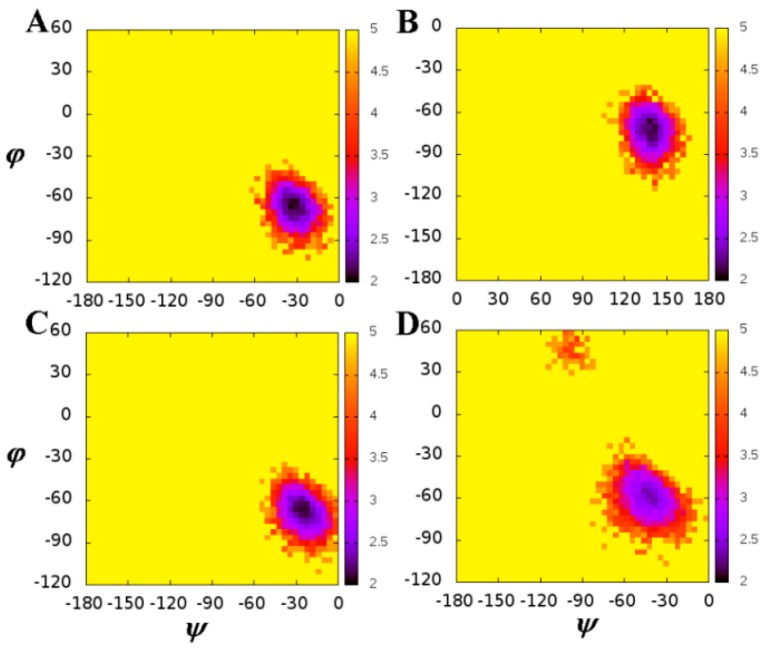
Contour maps of the free energies as a function of the backbone angles ψ and φ for residue 50 in WT (**A**,**B**) and I50V (**C**,**D**). (**A**) and (**C**) are in chain A, as well as (**B**) and (**D**) in chain B.

To evaluate the effect of mutations on the conformational change of the mutated residues, the free energy landscapes were constructed by using the backbone ψ and φ angles and shown in Figure 3, as well as in Figures S2–S4. We also calculated the averaged values of ψ and φ angles, as shown in Table 1. We noticed that the ψ and φ angles of residues in chain A are different from those in chain B in the WT complex, as well as in mutated complexes, especially for residue 50 (Figure 3). This suggests that the inhibitor bonds to the same residues of chains A and B with a different binding model. This is in accordance with the unsymmetric inhibitor and the analysis of RMSFs. So the conformational change caused by the mutation of PR should be analyzed according to different chains when the inhibitor is not *C*_2_-symmetry. We also noticed that the ψ and φ angles of residues in the WT system are different from those in mutated systems, especially for residue 50 of chain B (Figure 3B,D). The averaged value of ψ angles of residue 50 in chain B of WT and I50V systems are 133.36° and −42.28°, respectively. As residue 50 is located at the flap region of PR, the conformational change of backbone of residue 50 must lead to the steric redistribution of their nearby residues. Of course, the other mutations (D30N, I54M, and V82A) also cause the redistribution of their nearby residues. This conformational change would contribute to the GRL-0519 resistance.

### 2.2. Analysis of Binding Free Energy

To clarify the decrease of the binding affinity of four mutations relative to WT, the binding free energies were calculated for five complexes using the MM-PBSA method. Table 2 lists the components of the binding free energies. The calculated binding free energies are −23.79, −20.72, −19.65, −21.18, and −21.86 kcal/mol for WT, D30N, I50V, I54M, and V82A complexes, respectively. Although the calculated binding free energies are larger than the experimental data, the rank of the predicted binding free energies is in agreement with the experimental data. The differences of binding free energies between the experimental and the computational can be attributed to several sources [32], including the force field [33], solvation free energies, and entropic contribution. The polar solvation energies strongly depend on the radii and the non-polar solvation energies are represented by a linear relation to the solvent-accessible surface area (SASA) [21]. The conformational entropy is not considered. The van der Waals interactions (Δ*E*_vdW_) and the non-polar solvation energies (Δ*G*_nonpol_) are the basis for favorable binding free energies in all five complexes. The Δ*E*_vdW_ and Δ*G*_nonpol_ are responsible for the burial of GRL-0519’s hydrophobic groups upon binding. The favorable electrostatic term in the gas phase (Δ*E*_ele_) is opposed by the unfavorable polar solvation energies (Δ*G*_pol_). The resulting balance of the two contributions (Δ*G*_ele+pol_) of each mutated complex is more unfavorable than that of the WT complex.

We have performed the comparisons of the free energy components between the WT and each single mutation to elucidate the drug resistance mechanism. The Δ*G*_ele_ is obviously larger in the WT than in each mutation, especially in the D30N. The Δ*G*_vdW_ of D30N is the strongest among all five complexes. The enthalpic contribution of the WT is the largest among all complexes. Hence enthalpic contributions are the main factors for drug resistance. The entropic contribution also plays an important role in the I50V because the entropic contribution in I50V is obviously larger than in the WT.

### 2.3. Identification of the Key Residues Responsible for the Binding of Inhibitor

**Figure 4:**
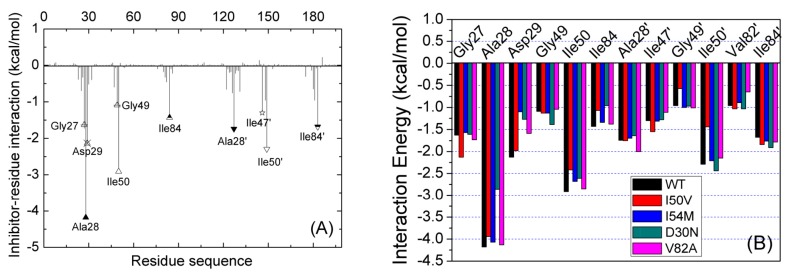
(**A**) Decomposition of Δ*G*_inhibitor-residue_ on a per-residue basis for the WT; (**B**) comparison of the binding free energy of the key residues for five complexes.

In order to find out which residue makes a significant intermolecular contribution to drug resistance, the interactions of GRL-0519 with individual residues were decomposed into the electrostatic interaction energy, van der Waals energy, and solvation free energy for all complexes and the results are depicted in Figure 4A and Figure S5. The decomposed approach is not only extremely useful to understand the drug-resistant mechanism at the atomic level, but also helps in selecting residues that are worth investigating [36,37]. From Figure 4 and Figure S5, we see that the interaction spectra of the five complexes are quite similar; the favorable contributions mainly come from 10 residues (Gly27, Ala28, Ala28′, Asp29, Ile47′, Gly49, Ile50, Ile50′, Ile84′, and Ile84) with |Δ*G*_inhibitor-residue_| ≥ 1.0 kcal/mol. Figure 4B shows the contribution of all key residues of the five complexes. The comparisons show that several residues contribute less in one complex relative to any other complex, such as Ala28 in D30N, Ile50′ and Gly49′ in I50V, and Asp29 in I54M. This implies that there is no large conformational change in the four mutated complexes compared with the WT complex as a whole except for some regions, such as the regions around the mutated residues.

**Figure 5:**
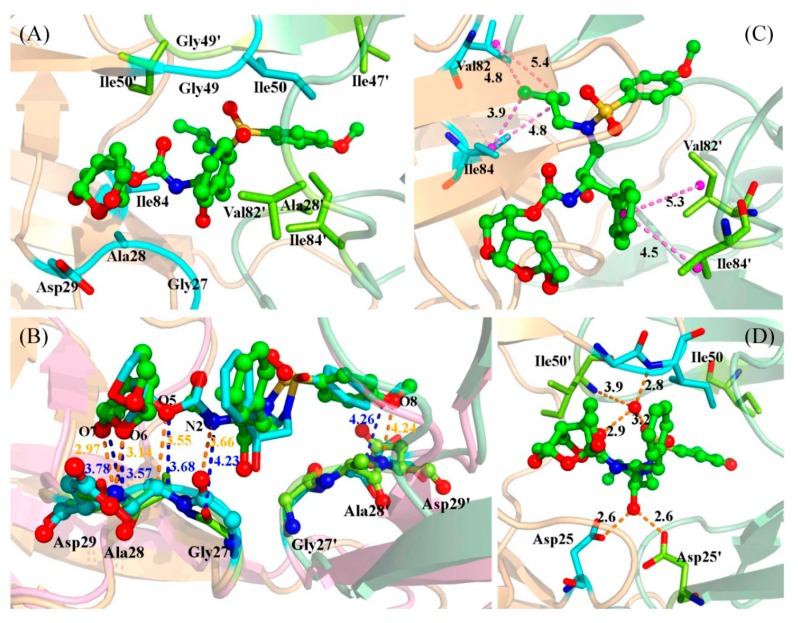
(**A**) Key residues interacted with the inhibitor in the WT; (**B**) Key residues of D30N superimposed on those of the WT. The averaged distances are shown with dashed lines in blue (D30N) and orange (WT). The D30N is shown in pink; (**C**) The van der Waals interactions between the inhibitor and residues in the WT. The averaged distances are shown with a dashed line; (**D**) The averaged distances between a bridge water molecule and Ile50/Ile50′/inhibitor, and the distances between the inhibitor and Asp25/Asp25′. Protein is shown in cartoon representation. Chain A is shown in light orange, and chain B in pale green. Key residues are shown in stick representation and labeled with residue names. The inhibitors are shown in stick and ball representation. The key distances are also labeled.

The key residues with large contribution can be divided into three sections in chain A, as well as in chain B. In a comparison of the contributions of sections between chain A and chain B, we found that there are two sections with large different contributions. We extracted the last snapshots of MD simulations for all systems to plot the figures. Figure 5A shows the residues of these two sections in the WT. As shown in Figure 4A and Figure 5A, one section around residue 28 in chain A interacted with group R1 of the inhibitor (see Figure 1B); its contribution in chain A is larger than in chain B, which interacts with group R4 of the inhibitor (see Figure 1B). The other section is around residue 82. This section in chain A interacts with group R3 of the inhibitor (see Figure 1B); its contribution in chain A is smaller than in chain B, which interacts with group R2 of the inhibitor (see Figure 1B). Hence, the inhibitor bonds to two chains with different binding models; group R1 can contribute larger free energy than group R4, and group R2 larger than group R3. So it may be a good strategy to replace group R2/R3 with group R1/R4 in the future when designing a potent new inhibitor of HIV-1 PR.

We have performed the comparisons of binding models of Gly27/Ala28/Asp29 between chain A and chain B of the WT. The contribution of Ala28 (−4.18 kcal/mol) is larger than that of Ala28′ (−1.27 kcal/mol). The methyl group of the side chain of Ala28 forms strong C–H...H–C interactions with the inhibitor, and the sum of the van der Waals energy and non-polar contributions are −2.47 and −1.84 kcal/mol for Ala28 and Ala28′, respectively. A C–H...O interaction was found between backbone atom CA of Ala28 and oxygen atom O5 of the inhibitor, with an averaged distance of 3.55 Å (Figure 5B, Table 3). As a result, the sum of the coulombic interaction and polar solvation free energies are −1.47 and 0.1 kcal/mol for Ala28 and Ala28′, respectively. As shown in Figure 5B and Table 3, the oxygen atom of the backbone of Gly27 forms a hydrogen bond with the nitrogen atom N2 of inhibitor with 38.63% occupancy and a distance of 3.21 Å. The nitrogen atom of the backbone of Asp29 forms two strong hydrogen bonds with oxygen atom O6 and O7 of group R1, with 76.29% and 98.74% occupancy, respectively (Table 3). On the other hand, the nitrogen atom of the backbone of Asp29′ forms a weak hydrogen bond with oxygen atom O8 of Group R4.

The binding free energy decomposition also shows that the residues Val82 and Ile84 in both chains A and B contribute favorably the van der Waals energies. The total binding free energies of Ile84 and Ile84′ are −1.68 and −1.4 kcal/mol, respectively. As shown in Figure 5C, the side chain of Ile84 interacted with group R3 by C–H...H–C interaction, as well as Ile84′ with groups R2 and R4 by C–H...πinteractions. The binding models of Val82/Val82′ are the same as that of Ile84/Ile84′. Hence the phenyl group is more favorable than the isopropyl group in the drug design.

A bridging water molecule forms a tetrahedral arrangement of hydrogen bonds connecting the amide nitrogen atoms of Ile50/Ile50′ with the sulfonamide oxygen and the carbamate carbonyl oxygen of inhibitor. The calculated binding free energy by MM-PBSA does not include the energy from the water bridge, for the water molecule was stripped in the process of MM-PBSA. The free energy decomposition has demonstrated that the Ile50 and Ile50′ contribute large favorable van der Waals energies. As shown in Figure 5D, the side chains of Ile50 and Ile50′ interact with groups G3 and G4 of the inhibitor. The side chain of Ile47′ also interacts with group G4 by C–H...π interaction. Although the binding free energies of Asp25 and Asp25 are small, they show very large electrostatic interaction because they form hydrogen bonds with oxygen atom O3 of the inhibitor with high occupancy (Figure 5D and Table 3).

### 2.4. Mutations’ Influence on the Binding of Inhibitor

The hydrogen bond between oxygen atom O6 of the inhibitor and the nitrogen atom of the backbone of residue 30 in chain A of the WT with 90.4% occupancy is stronger than in D30N complex with 77.4% occupancy (Table 3). However, the decomposition of free energy shows that the electrostatic contribution of residue 30 in D30N is more favorable than in the WT. This can be explained by the large attraction of unlike charges because group R1 has positive charge and the side chain of Asp30 has negative charge. The drug resistance of D30N mutation comes from the weak binding affinity for residues 28 and 29 of chain A in D30N relative to the WT. As shown in Figure 5C, the key distances, which can symbolize the binding between residues and inhibitor, are larger in D30N than in the WT. We can say that the replacement of Asp30 with Asn30 increases the binding affinity of residue 30 and decreases the binding affinity of residues 28 and 29.

**Figure 6:**
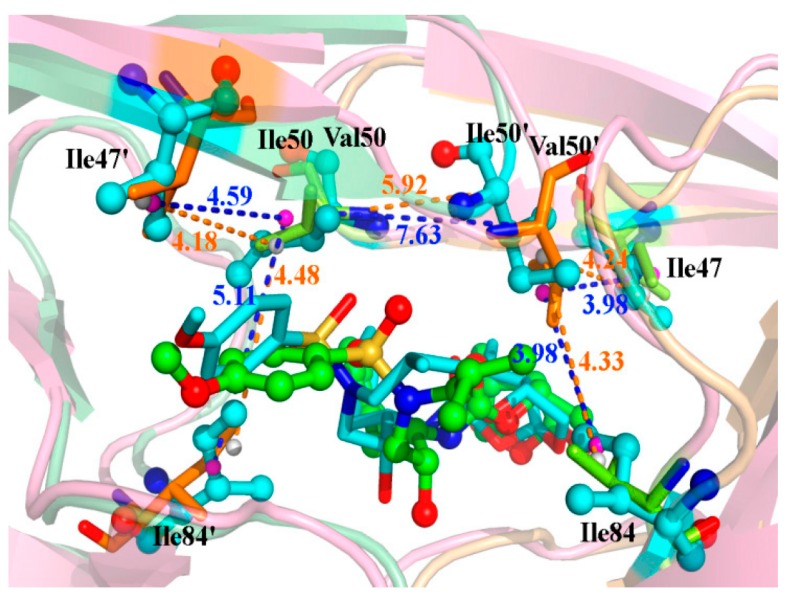
The key residues of the I50V complex, superimposed on those of the WT complex. The averaged distances are shown with dashed lines in blue (I50V) and orange (WT). The representation is the same as in Figure 5.

The distances between two carbon atoms CA of residue 50 in both chains are 6.00 and 6.09 Å in the crystallographic structures of WT and I50V, respectively. This implies that mutations Ile50 to Val50 hardly affect the relative location of residues around Val50. However, the averaged distances over the last 40 ns of MD are 5.92 and 7.63 Å for the WT and I50V complexes, respectively (Figure 6). This implies that the mutation would affect the conformation of residues around mutated residues. The hydrogen bond analysis shows that the hydrogen bonds between the bridging water molecule and these two residues in I50V complex are weaker than in the WT complex (Table 3). To show the conformational change, we calculated the molecular mechanic energies and the minimum distances between residue 50 and its nearby residues 84 and 47 in the corresponding chain (Table 4). The free energy of residue 50 in chain A of the WT complex is more favorable than in the I50V complex, but not in chain B. On the other hand, the minimum distances between the heavy atoms of the two side chains also show similar results. The minimum distances in the WT complex are larger than in the I50V complex for residue in chain A and *vice versa* in chain B. This can be explained by the stable hydrophobic core formed by group G2 of the inhibitor and the side chains of residues 50′ and 84. The hydrophobic interaction makes Val50′ move to Ile84 and Ile47, filling the space caused by mutation. So the distances between the two CA atoms of Val50 and Val50′ become larger in the I50V complex relative to in the WT complex.

**Figure 7:**
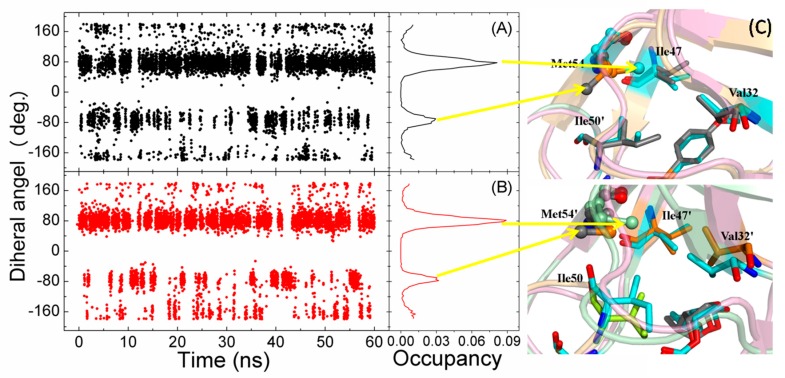
Dihedrals of the four heavy atoms of the side chain of residues 54 in chain A with black line (**A**) and in chain B with red line (**B**); (**C**) The major and the minor conformations are shown, and the heavy atoms of the side chain of residue 54 are shown in ball representation. The representation is the same as in Figure 5.

The Met54′ showed two alternative conformations (major and minor) in the crystallographic structure of I54M. The two conformations were observed for both Met54′ and Met54 in the MD simulation of I54M. To monitor the conformational change of Met54, the dihedrals of four heavy atoms of the side chain of Met54 and Met54′ were calculated and shown in Figure 7. It is easy to find the major and minor conformations in both Met54 and Met54′. The major conformation was the methyl point at the hydrophobic core formed by the side chains of Val32, Ile47, and Ile50′, and the minor conformation with the methyl in opposite direction. There is a conformation with the dihedrals at 180 degrees. This conformation would be the intermediate state between the major and minor. The residues around residue 54 form a hydrophobic core. To show the influence by the mutation of residue 54, we have calculated the averaged RMSDs of the heavy atom of the hydrophobic core in I54M (0.78 Å) and in the WT (0.68 Å). This implies that the hydrophobic core in I54M becomes flexible relative to the WT complex. The hydrogen bond between the backbone nitrogen atom of Asp29 and the O6 atom of the inhibitor in I54M is weaker in the WT (Table 3). The interaction energy of Asp29 is obviously weaker in I54M than in the WT.

Val82/Val82′ mainly interact with R3/R2 by the C–H...H–C or C–H...π interactions. The isopropyl group of residue 82 in the WT complex is replaced by a methyl in the V82A complex. The side chains of Ala82/Ala82′ in the V82A complex are smaller and lead to the loss of van der Waals interaction compared with those of Val82/ Val82 in the WT complex.

## 3. Materials and Methods

### 3.1. System Setups

Atomic coordinates of the five complexes studied in this work were obtained from the Protein Data Bank (PDB). The PDB IDs are 3OK9, 4HDB, 4HDP, 4HE9, and 4HDF for WT, D30N, I50V, I54M, and V82A, respectively [10]. A bridge water molecule plays important roles in the binding between PR and inhibitors. This water molecule is observed in almost all PR-inhibitor complexes [38,39] Hence, we kept the crystallographic water molecules within 5 Å of PR in the starting model. Two catalytic aspartic acids of PR can form hydrogen bonds with the hydroxyl of the inhibitor. To form hydrogen bonds easily, only one of these two residues was considered to be protonation. In this work, a proton was added to oxygen atom OD2 of Asp25, in accordance with other works [31,40].

The protein parameters used the AMBER force field (FF03) [41]. The force field for the inhibitor does not exist [42], so we developed it. The inhibitor was first optimized at the HF/6-31G* level with the Gaussian 03 [43]. The partial atomic charges were fitted by the Restrained Electrostatic Potential (RESP) procedure [44]. The general AMBER force field (GAFF) [45] parameters and the partial charges were assigned using the ANTECHAMBER module in the AMBER 12 package. The TIP3P water model was used to dissolve the complex with a truncated octahedron periodic box extended to a distance of 10 Å from solute atoms [46]. To neutralize the charges of systems, an appropriate number of chloride counterions were added.

### 3.2. Molecular Dynamics Simulations

The AMBER 12 package was used to do all simulations [47]. Particle-mesh Ewald (PME) method was used to treat the long-range electrostatic interactions [48]. The SHAKE algorithm was employed for all atoms covalently bonded to a hydrogen atom [49]. The time step was set at 2 fs with a cutoff of 12 Å. The Sander module in the AMBER 12 package [47] was used to do the minimization and MD simulation. To relieve the bad contacts in the initial structure, we carried out a two-step extensive energy minimization process based on the steepest descent method followed by the conjugate gradient algorithm. After energy minimization, each system was gently heated from 0to 300 K over a period of 70 ps. All solute atoms were constrained with a constant force of 2 kcal/(mol·Å^2^) at constant volume. Subsequently, each system was adjusted so the solvent density was 90 ps at constant pressure. Finally, 60-ns simulations without any restrictions were performed at constant pressure. The Langevin thermostat was employed to regulate the temperature at 300 K. The pressure was kept at 1.0 atm. The ptraj module of Amber Tools software [50] was used for RMSD, RMSF, dihedral, and hydrogen bond analysis every 2 ps.

### 3.3. MM-PBSA Calculations

Strictly, the averaged free energy terms should be estimated from three separate simulations of the complex, the free ligand, and the HIV protease [51]. In the one-average MM-PBSA, the change in structure of the ligand and the receptor upon binding was ignored; however, it requires fewer simulations, improves precision simulation, and leads to a cancellation of the binding free energy (Δ*G*_bind_) [18]. So the one-average MM-PBSA is more commonly used than the separate simulation. In this work, we calculated the binding free energies by using a one-average MM-PBSA method, which is supplied with AMBER 12 package [47]. In the MM-PBSA method, Δ*G*_bind_ is divided into enthalpic (Δ*H*) and entropic (−TΔS) contributions:

(1) $$\Delta G_{bind}=\Delta H-T\Delta S$$

The Δ*H* is divided into the gas-phase molecular mechanics energy (Δ*E*_MM_) and the solvation free energy (Δ*G*_solv_):

(2) $$\Delta H=\Delta E_{MM}+\Delta G_{solv}$$

The Δ*E*_MM_ is composed of a noncovalent van der Waals component (Δ*E*_vdW_) and an electrostatic energies component (Δ*E*_ele_). The Δ*G*_solv_ is further divided into a polar (Δ*G*_pol_) and a nonpolar part (Δ*G*_nonpol_):

(3) $$\Delta E_{MM}=\Delta E_{vdw}+\Delta E_{ele}$$

(4) $$\Delta G_{solv}=\Delta G_{pol}+\Delta G_{nonpol}$$

The Δ*G*_pol_ is calculated with the PBSA module of the AMBER suite. The Δ*G*_pol_ is determined by equation Δ*G*_nonpol_ = γSASA + β. The empirical constant γ was set to 0.005 kal/(mol·Å^2^) [52]. The SASA is determined by the LCPO method [53]. The normal-mode analysis was used to calculate the entropic contribution with the NMODE module [54]. Two hundred snapshots taken from the last 40 ns (21–60 ns) of the MD simulations at an interval of 200 ps were applied to calculate the entropic contribution. Δ*E*_MM_ and Δ*G*_solv_ were averaged on 800 snapshots, which were extracted from the last 40 ns of MD trajectories at intervals of 50 ps.

## 4. Conclusions

In this work, we carried out five 60-ns MD simulations to investigate the stability and dynamics of GRL-0519 in the WT and four mutation complexes. We calculated the binding free energy between GRL-0519 and HIV-1 PR using the MM-PBSA method. The ranking of calculated binding free energies is in accordance with the experimental data. Our results show that the main factor is enthalpic contribution for the drug resistance. The entropic contribution also plays an important role in the I50V complex. The decomposition of inhibitor–residue interactions and the structural analysis have demonstrated that the residues of the two chains have different binding models for the GRL-0519 *C*_2_-unsymmetry. Groups R1 and R4 form hydrogen bonds with the inhibitor. Groups R2 and R3 mainly contribute van der Waals energies by C–H…π and C–H…H–C interactions. The conformational changes were only found for the residues around the mutated residues. In the D30N complex, the binding affinity of residue 30 increases but that of residues 28 and 29 decrease. The I50V mutation changes the binding package. Residue 54 in the I54M complex shows two alternate conformations. The determined structure–affinity relationship may be helpful in the future design of a potent inhibitor of HIV-1 PR and gives insights into the nature of mutational effect.

## Figures and Tables

**Table 1:** The averaged backbone angles of mutated residues.

Residues	Chains	WT		Mutation	
		φ (°)	ψ (°)	φ (°)	ψ (°)
30	A	−115.63	149.32	−125.53	153.97
	B	−127.02	152.80	−135.51	150.64
50	A	−66.61	−31.90	−66.88	−24.81
	B	−69.57	133.36	−56.66	−42.28
54	A	−135.28	152.63	−134.94	139.37
	B	−132.16	147.75	−141.02	137.87
82	A	−144.86	149.19	−149.81	152.56
	B	−145.22	152.89	−148.01	150.60

**Table 2:** Energies for binding of GRL-0519 to WT and mutations by the MM-PBSA method.

Compounds	WT		D30N		I50V		I54M		V82A	
Items ^a^	Mean	σ ^b^	Mean	σ ^b^	Mean	σ ^b^	Mean	σ ^b^	Mean	σ ^b^
Δ*E*_ele_	−58.49	0.19	−41.66	0.21	−49.89	0.26	−56.59	0.20	−58.12	0.21
Δ*E*_vdw_	−69.18	0.12	−73.02	0.11	−70.99	0.13	−70.06	0.13	−68.84	0.13
Δ*G*_nonpol_	−7.44	0.00	−7.55	0.00	−7.36	0.00	−7.54	0.00	−7.51	0.00
Δ*G*_pol_	84.09	0.18	74.23	0.17	79.47	0.18	86.11	0.16	85.72	0.17
Δ*G*_ele+pol_	25.60	0.19	32.57	0.19	29.58	0.22	29.52	0.18	27.60	0.19
Δ*G*_vdw+nonpol_	−76.62	0.06	−80.57	0.06	−78.35	0.06	−77.60	0.07	−76.35	0.06
Δ*H*	−51.02	0.15	−48.00	0.15	−48.77	0.17	−48.08	0.16	−48.75	0.15
TΔ*S*	−27.23	0.48	−27.28	0.40	−29.12	0.41	−26.90	0.46	−26.89	0.46
Δ*G*_bind_	−23.79	–	−20.72	–	−19.65	–	−21.18	–	−21.86	–
Δ*G*_exp_^c^	−12.77	–	−11.05	–	−10.31	–	−11.61	–	−11.66	–
ΔΔ*G*_bind_	–	–	−3.07	–	−4.14	–	−2.61	–	−1.93	–
ΔΔ*G*_exp_	–	–	−1.72	–	−2.46	–	−1.16	–	−1.11	–

^a^ The symbols of the energy terms are described in the section on MM-PBSA calculations. All values of energies are given in kcal/mol; ^b^ Standard errors were calculated by σ = standard deviation/N^1/2^ [29,34,35]; ^c^ The experimental binding free energies (Δ*G*_exp_) were calculated from published inhibition constants (*K*_i_) by equation Δ*G*_exp_ = −*TlnK*_i_ [32], where *R* is ideal gas constant, *T* is temperature in K (300 K is used in this paper), and the *K_i_* values were obtained from the IC_50_ values estimated from an inhibitor dose–response curve [10]. ΔΔ*G* = Δ*G*_WT_ − Δ*G*_Mutation_.

**Table 3:** Main hydrogen bonds involved in the GRL-0519 binding pocket.

Hydrogen Bond ^a^	WT		D30N		I50V		I54M		V82A	
	Occ (%)	Dist (Å)	Occ (%)	Dist (Å)	Occ (%)	Dist (Å)	Occ (%)	Dist (Å)	Occ (%)	Dist (Å)
Asp25′@OD1···GRL@H17–O3	100.00	2.62	59.54	2.65	46.43	2.65	49.60	3.14	53.26	3.03
Asp25′@OD2···GRL@H17–O3	50.57	3.07	37.11	2.71	63.17	2.78	100.00	2.62	99.40	2.64
GRL@O3···Asp25@HD2–OD2	99.97	2.64	–	–	31.86	2.75	99.80	2.65	96.37	2.64
Wat@O···Ile50′@H–N	81.83	3.09	84.46	3.02	43.03	3.07	86.63	3.04	78.63	3.13
Wat@O···Ile50@H–N	98.94	2.97	94.86	3.09	91.89	3.08	99.17	2.96	98.91	2.98
GRL@O4···Wat@H1–O	95.37	2.76	79.03	2.75	88.94	2.75	99.23	2.75	78.94	2.78
GRL@O2···Wat@H2–O	89.23	2.82	77.69	2.79	88.57	2.85	96.43	2.77	68.40	2.87
Gly27@O···GRL@H19–N2	38.63	3.21	21.91	3.23	60.54	3.15	34.80	3.19	50.11	3.12
GRL@O5···Ala@HA-CA	40.40	3.39	27.83	3.39	–	–	40.09	3.39	36.37	3.39
GRL@O7···Asp29@H–N	98.74	2.96	71.11	2.99	96.71	2.99	99.00	2.98	96.97	3.00
GRL@O6···Asp29@H–N	76.29	3.10	29.06	3.20	88.54	3.05	56.09	3.15	75.63	3.10
GRL@O8···Asp30′@H–N	27.37	3.24	26.14	3.28	33.80	3.27	–	–	46.83	3.21
GRL@O6···Asp30@H–N	90.40	3.14	77.40	3.17	80.34	3.16	75.20	3.20	71.94	3.19

^a^ The hydrogen bonds are determined by the following criteria. The distance (Dist) between acceptor and donor is less than 3.5 Å, and the angle donor-H···acceptor is greater than or equal to 120°. Occupancy (Occ) is defined as the percentage of simulation time that a specific hydrogen bond exists.

**Table 4:** The distances and energies between residues 50 and 84/47.

Residues	Distance (Å)		Energy (kcal/mol)	
	WT	I50V	WT	I50V
Ile(Val)50′-Ile84	4.33	3.98	−0.54	−0.55
Ile(Val)50′-Ile47	4.24	3.98	−0.59	−0.40
Ile(Val)50-Ile84′	4.48	5.11	−0.38	−0.17
Ile(Val)50-Ile47′	4.18	4.59	−0.60	−0.44

## Supplementary Materials

Supplementary materials can be found at http://www.mdpi.com/1422-0067/17/6/819/s1.
