# Supplementary material for: Computational Studies of a Mechanism for Binding and Drug Resistance in the Wild Type and Four Mutations of HIV-1 Protease with a GRL-0519 Inhibitor
Source: Int J Mol Sci. 2016 May 27;17(6):819. doi: 10.3390/ijms17060819 (PMC4926353; doi:10.3390/ijms17060819)
Supplement: Supplementary file 1 [file ijms-17-00819-s001.pdf]

Supplementary Materials: Computational Studies of a Mechanism for Binding and Drug Resistance in the Wild Type and Four Mutations of HIV-1 Protease with a GRL-0519 Inhibitor

Guodong Hu, Aijing Ma, Xianghua Dou, Liling Zhao and Jihua Wang

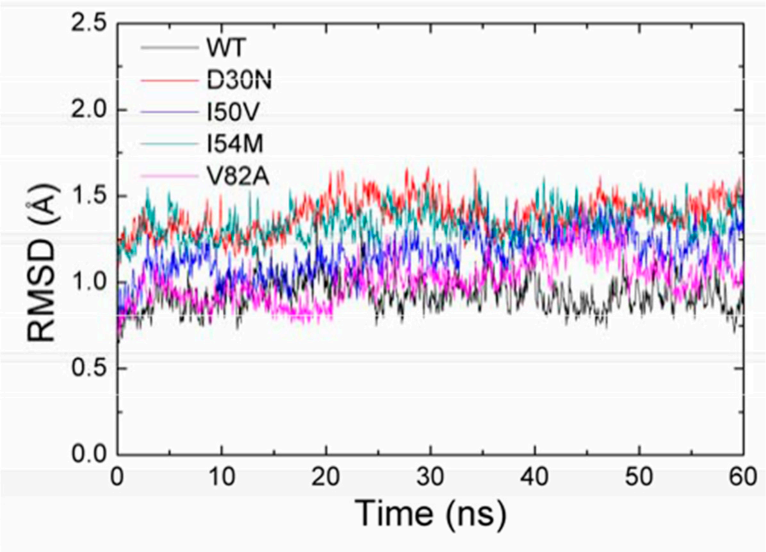

Figure S1. The root-mean-square deviations (RMSDs) of the back bone atoms relative to the crystal structure of the WT as a function of MD time.

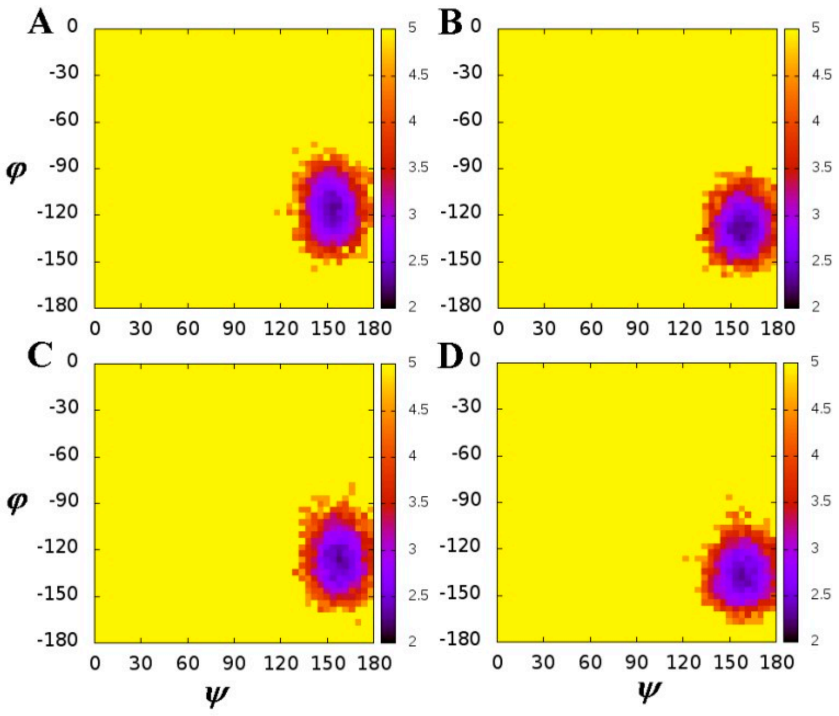

Figure S2. Contour maps of the free energy as a function of the backbone angles ψ and φ for residue 30 in the WT (**A**,**B**) and D30N (**C**,**D**). (**A**) and (**C**)are in chain A, and (**B**) and (**D**) in chain B.

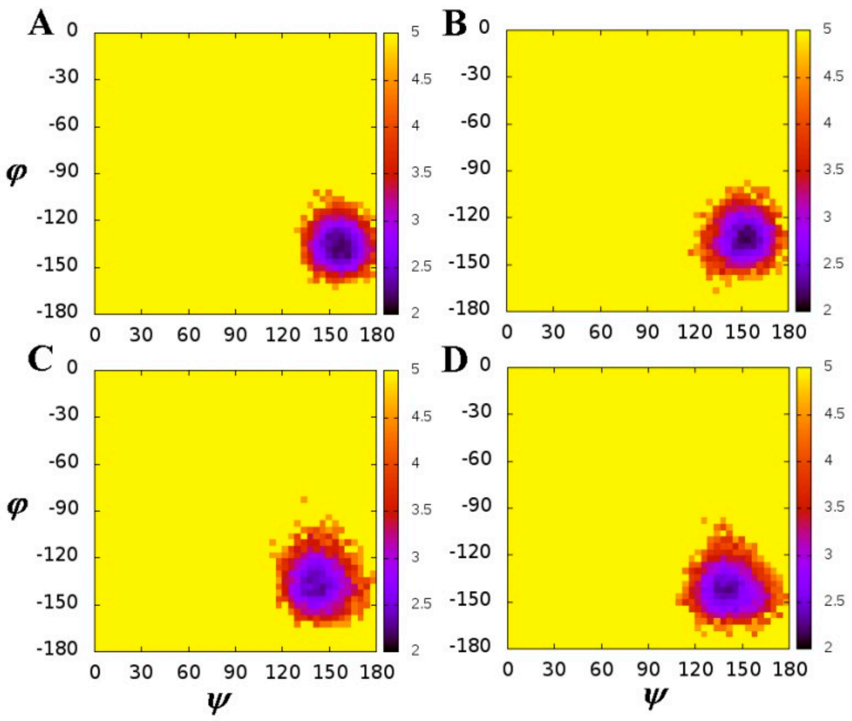

Figure S3. Contour maps of the free energy as a function of the backbone angles ψ and φ for residue 54 in the WT (**A**,**B**) and I54M (**C**,**D**). (**A**) and (**C**)are in chain A, and (**B**) and (**D**) in chain B.

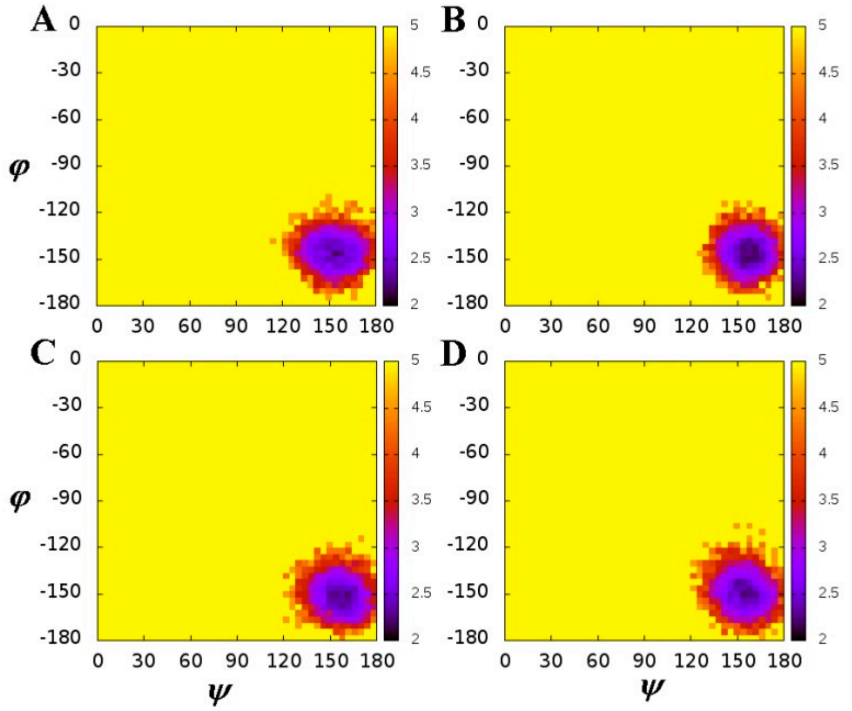

Figure S4. Contour maps of the free energy as a function of the backbone angles ψ and φ for residue 82 in the WT (**A**,**B**) and V82A(**C**,**D**). (**A**) and (**C**)are in chain A, and (**B**) and (**D**) in chain B.

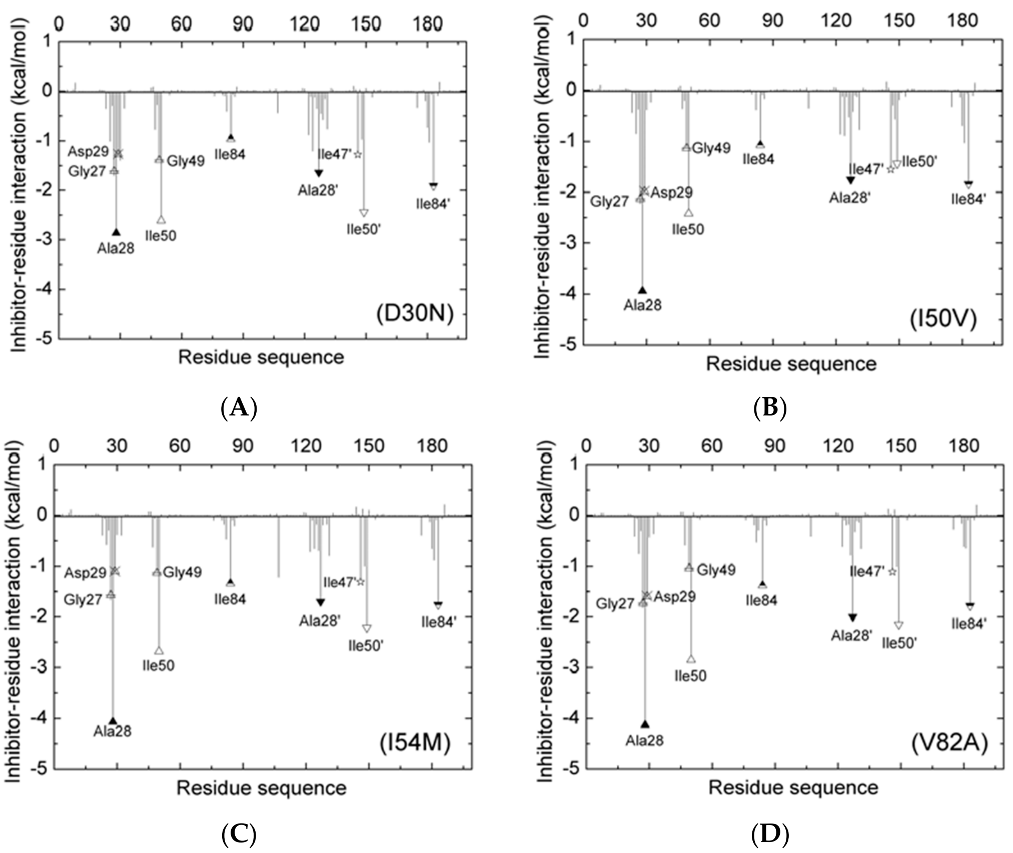

Figure S5. (**A**–**D**) Decomposition of $\Delta G_{\text{inhibitor–residue}}$ on a per-residue basis for D30N, I50V, I54M, and V82A.
